# Recent Development of the Laser Interferometer for Taiji Space Gravitational Wave Detection

**DOI:** 10.34133/research.1252

**Published:** 2026-04-16

**Authors:** Heshan Liu, Juan Wang, Wei Tao, Keqi Qi, Shaoxin Wang, Ruihong Gao, Pan Li, Peng Dong, Wei Sha, Ziren Luo, Wenrui Hu

**Affiliations:** ^1^ National Microbiology Laboratory, Institute of Mechanics, Chinese Academy of Sciences, Beijing, China.; ^2^ Changchun Institute of Optics, Fine Mechanics and Physics, Chinese Academy of Sciences, Changchun, China.; ^3^ School of Fundamental Physics and Mathematical Sciences, Hangzhou Institute for Advanced Study, UCAS, Hangzhou, China.

## Abstract

In this study, we have designed a full-function interferometer OB (optical bench) for the Taiji program and, for the first time, provided detailed parameter specifications for its interferometer system. Additionally, we constructed a first-generation Taiji interferometer OB and ground test system and conducted preliminary testing and calibration. A comprehensive analysis was carried out on the various noise sources impacting the interferometer system, including laser frequency noise, laser power noise, thermal drift noise, and angular jitter noise. The results revealed that the noise level of the interferometer system reached 1.1 pm/Hz^1/2^ at 1 Hz, 5 pm/Hz^1/2^ at 0.1 Hz, and 8 pm/Hz^1/2^ at 0.01 Hz; these levels satisfied the requirements of the Taiji-2 mission. This work marks the first step in transitioning the Taiji program’s interferometer system from a principle prototype to an engineering prototype, playing a critical role in its future development.

## Introduction

The Taiji program is an SGWD (space gravitational wave detection) mission proposed by the Chinese Academy of Sciences [1,2]. The mission is composed of 3 satellites, which form an equilateral triangle with a distance of 3 million km between each satellite. It utilizes a laser interferometer to detect the small displacements induced by gravitational waves at the level of pm/Hz^1/2^ between the test masses (TMs), within a frequency band of 0.1 mHz to 1 Hz [3–5]. Based on the 3-phase implementation strategy of the Taiji program, 3 sequential missions—Taiji-1 through Taiji-3—are planned. Taiji-1 and Taiji-2 are primarily dedicated to in-orbit validation of key technologies. To date, the Taiji-1 single-satellite validation mission has been successfully completed, and the focus has now shifted to critical technology development for Taiji-2. Meanwhile, the Taiji program is actively seeking additional funding to support an upgraded Taiji-2 mission, which aims to deploy a full 3-satellite constellation to verify all relevant technologies. This enhanced Taiji-2 mission would differ from Taiji-3 only in terms of measurement precision and mission duration. The other SGWD missions also include LISA [6,7] and Tianqin [8,9], which have the same constellation configuration but different arm lengths and orbits. Many technologies have been developed for the ground demonstration of SGWD optical systems for different missions; these technologies include laser interferometers [6,9–11], phasemeters [12,13], weak-light phase locking [14–16], DWS (differential wave-front sensing) [17,18], and laser beam acquisition [19,20].

To date, the technical verification satellites for SGWD that have been launched include LISA Pathfinder [21,22], GRACE follow on [23], Taiji-1 [24], and Tianqin-1 [25]. The LISA Pathfinder successfully demonstrated the function and sensitivity of the OB (optical bench), and a sensitivity of 35 fm/Hz^1/2^ was attained. The GRACE follow on initially demonstrated the intersatellite laser interferometer at several hundred km at the nm level. Similar with the LISA Pathfinder, Tianqin-1 demonstrated a laser interferometer with a sensitivity of 30 pm/Hz^1/2^ in the frequency range of 0.1 to 1 Hz. The interferometer of Taiji-1 could also reach a sensitivity of 30 pm/Hz^1/2^ in the frequency range of 0.01 to 10 Hz. In contrast, the KZ-1A rocket had a lower mechanical strength than the liquid rocket. These results subsequently indicated that the OB of Taiji-1 could encounter more severe mechanical stresses than that of the LISA Pathfinder. Consequently, the traditional optical-mechanical structure has been favored over optical bonding for the implementation of the OB of Taiji-1 [26].

However, the majority of current OB designs are planar structures, which cannot meet the installation requirements of the final SGWD telescope and inertial sensor. Recently, the prevailing design approach is the adoption of a 3-dimensional optical path design, with corresponding devices installed both on the front and back of the OB, as shown by the Taiji and LISA programs [11,27]. The OB is installed vertically and connected to the telescope and inertial sensor through laser beams perpendicular to the optical platform. Unfortunately, these 3-dimensional optical path designs have higher requirements for the construction of the OB, and currently, no reports of successful implementations are available. Based on this, this paper presents the design of a first-generation interferometer OB for the Taiji program, for which detailed parameter specifications have been provided for the first time. Furthermore, the OB and a ground test system have been constructed, and preliminary testing and calibration have been conducted.

## Results and Discussion

### Requirements of Taiji program

In the Taiji program [28], the baseline design parameters are listed in Table 1.

**Table 1.** Baseline design parameters for Taiji program

	Arm length	Laser power	Telescope diameter	Displacement noise	Acceleration noise
Taiji-3	3 × 10^9^ m	2 W	0.4 m	8 pm/Hz^1/2^	3 × 10^−15^ ms^−2^/Hz^1/2^
Taiji-2	3 × 10^9^ m	2 W	0.4 m	30 pm/Hz^1/2^	3 × 10^−14^ ms^−2^/Hz^1/2^

**Fig. 1.**
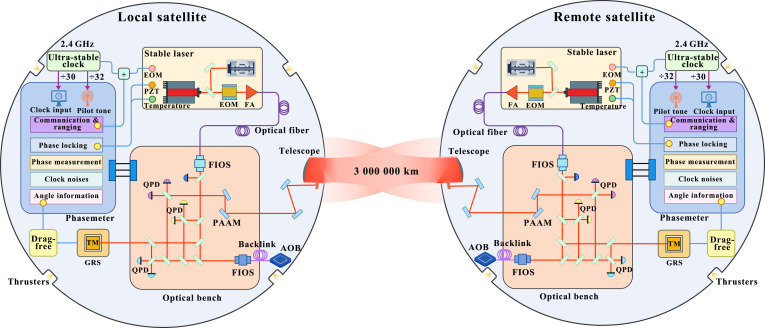
The composition and interconnection of the Taiji interferometer system. EOM, electro-optic modulator; FA, fiber amplifier; GRS, gravitational reference sensor; FIOS, fiber injector optical subassembly; PAAM, point-ahead angle mechanism; AOB, adjacent optical bench.

The most important function of the interferometer is to measure the displacement fluctuation between the 2 TMs. The requirement of the interferometer is 8 pm/Hz^1/2^ at a distance of 3 million km in the frequency range of 0.1 mHz to 1 Hz. The interferometer system consists of a stable laser, an OB, a QPD (quadrant photodiode detector), a phasemeter, and a telescope. The composition and connection relationship of each part are shown in Fig. 1.

The ultrastable laser produces a laser beam, with high frequency and power stabilities. The laser beam is sent to the OB and guided by different functional optical mirrors and lenses to form different interferometers for various purposes. At the same time, a portion of the laser beam is transmitted through the telescope to the remote spacecraft. The beam is locked at the nW level in the remote satellite and is returned; the returned beam is received by the telescope and enters the OB. The interference signal is received by the photodetector and converted into an analog electrical signal. Finally, the analog signal is processed by the phasemeter, and the displacement variation information is output for further analysis. The detailed functions and requirements of the interferometer system are as follows:

1. Readout of the displacement variations between the TMs with pm/Hz^1/2^ precision.

2. Intersatellite laser link acquisition and beam pointing [20].

3. Optical readout for the drag-free control system to provide displacement variation and angular jitter between the TM and the local OB.

4. Intersatellite absolute laser ranging and data communication [29].

5. Intersatellite clock noise transmission [30].

6. Weak-light phase locking and arm locking [31].

The main functions of the interferometer system are measuring the displacement variations and angular jitter between the TMs of the intersatellite and single satellite. Moreover, the intersatellite interferometer requires the integration of additional functionalities, including intersatellite laser communication, absolute ranging, transmission of clock noise between satellites, phase locking under nW conditions, and arm locking. The main indicators and requirements of the Taiji interferometer system are listed in Table 2.

**Table 2.** The key indicators and requirements of the Taiji interferometer

Functions	Indicators	Requirements	
		Taiji-2	Taiji-3
Intersatellite displacement and angle measurement; laser beam pointing; TM readout	Science interferometer	≤30 pm/Hz^1/2^	≤8 pm/Hz^1/2^
	TM/reference interferometer	≤10 pm/Hz^1/2^	≤1 pm/Hz^1/2^
	Angle measurement	≤10 nrad/Hz^1/2^	≤1 nrad/Hz^1/2^
Intersatellite laser data communication; absolute ranging	Communication rate	≥20 kbps	≥20 kbps
	Ranging accuracy	≤10 m	≤1 m
Intersatellite clock noise transfer	Noise	≤10 pm/Hz^1/2^	≤1 pm/Hz^1/2^
Weak-light phase lock	Noise	≤6 pm/Hz^1/2^	≤6 pm/Hz^1/2^
Arm-locking	Noise	≤10^−4^ Hz/Hz^1/2^	≤10^−4^ Hz/Hz^1/2^

In addition to the above requirements for the specified parameters, the interference system also needs to integrate PAAM (point-ahead angle mechanism), laser acquisition cameras, and beam monitoring cameras. Therefore, the OB needs to be designed to meet the requirements of the multi-functions.

### Optical layout

**Fig. 2.**
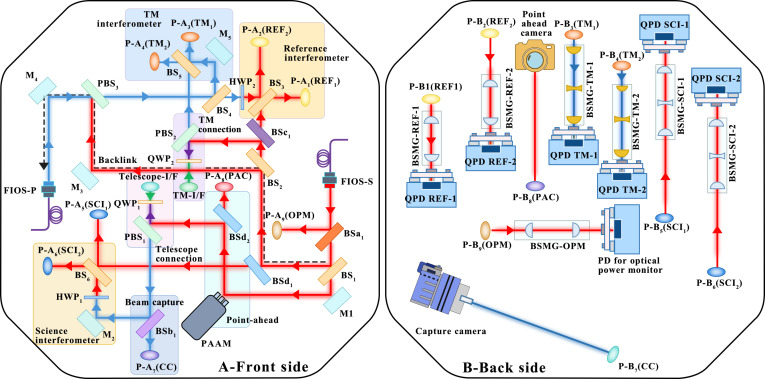
Optical layout design of the first-generation optical bench for the Taiji program. Almost all the mirrors were installed on the A-front side which faced to telescope, and all the photo-receiver were installed on the B-back side which faced to the inertial sensor. The lines of different colors represent lasers with different polarization states. Blue indicates a linearly polarized laser parallel to the plane of the optical bench; red indicates linearly polarized light perpendicular to the plane of the optical bench; and green indicates that the linearly polarized light becomes elliptically polarized after quarter wave plate (QWP). The purple line indicates that the laser path represented by blue coincides with that represented by red. M, mirror; BS, beam splitter (R/T: 50/50); BSa, beam splitter (10/90); BSb, beam splitter (90/10); BSc, beam splitter (1/99); BSd, beam splitter (99/1); PBS, polarizing beam splitter; FIOS, fiber injector optical subassembly; P-A, periscope in side A; P-B, periscope in side B; HWP, half wave plate; Telescope-I/F, telescope interface; TM-I/F, test mass interface; PAAM, point ahead angle mechanism; BSMG, beam shrinking mirror group; QPD, quadrant photodiode.

The optical layout of the OB is shown in Fig. 2. This optical path layout features passive optical components on the front and heating devices (such as QPDs and cameras) on the back. This layout effectively isolates the impact of the heating devices on the stability of the optical path. Additionally, it minimizes stray light caused by the secondary reflections of the optical components into the detector, thereby reducing the noise level of the OB. The front side of the OB faces the telescope, and the back side faces the inertial sensor. Telescope-I/F and TM-I/F are the interfaces between the OB and them for laser transmission. The laser is transmitted between the front and back of the OB through the periscopes.

The OB includes 3 interferometers and 3 auxiliary optical paths. They are the reference interferometer (received by the QPD-REF), the TM interferometer (received by the QPD-TM), the science interferometer (received by the QPD-SCI), the intersatellite capture optical path (from Telescope-I/F to the capture camera), the point-ahead optical path (from PAAM to Telescope-I/F), and the backlink optical path (from FIOS-s to FIOS-p). To ensure sufficient laser intensity for both the reference interferometer and the TM interferometer, the majority of the laser power is emitted to its greatest extent. Therefore, several beamsplitter ratios are used. The detailed functional introductions of the different optical paths are provided below.

Reference interferometer: (a) Common mode noise rejection between the interferometers. (b) Providing sensing signals for the phase locking of 2 lasers within the satellite.

TM interferometer: (a) Readout of the motion of the TM between the local TM and the local OB, including translation and rotation. (b) Providing sensing signals for the drag-free control of satellites.

Science interferometer: (a) The readout of the satellite’s motion between the local satellite and the remote satellite, including the translation and rotation. (b) Providing sensing signals for weak-light phase locking between the 2 satellites and arm locking. (c) Auxiliary functions of the laser link including absolute laser ranging, data communication, and clock noise transfer. By phase-modulating the laser beam, the communication codes and clock noise are transmitted between the satellites through the sideband of the laser.

Intersatellite laser link acquisition: Accurately establishing an intersatellite laser interferometric link and ensuring interference contrast are prerequisites for laser interference measurement. The Taiji program requires that the relative disturbance of the angle between the intersatellite laser beams is better than 10 nrad/Hz^1/2^. Currently, this is achieved through 3 steps: star sensor (range/accuracy: 0.35 rad/5 μrad), laser beam capture (200 μrad/40 nrad), and laser pointing (1.5 μrad/10 nrad/Hz^1/2^). The laser link acquisition mentioned here is the second stage, where the satellite scans an uncertain area under control to receive the laser beam and initially establishes an interferometric signal. Finally, through the DWS technique of the interferometric signal, the angle is further measured, and laser pointing control is performed to achieve 10 nrad/Hz^1/2^ accuracy. Laser link acquisition on the OB mainly involves a CCD (charge-coupled device) that monitors the position of the received laser beam.

Beam point ahead: Due to the distance of 3 million km between the 2 satellites, the laser beam takes 10 s to reach the remote spacecraft. However, relative tangential motion occurs between the satellites; thus, the current pointing direction cannot accurately reflect the position of the laser beam 10 s later. Therefore, a measuring target of 10 s in the future is needed. This function involves a PAAM that controls the angle of the outgoing beam and a CCD detector that monitors the angle of the laser beam.

Backlink: This connects the 2 local OBs in one satellite to reduce the number of lasers. Based on the principle of polarization multiplexing [32], the laser beam on one OB serves both as a measuring beam for laser measurements and as a reference beam for another OB.

### Experimental setup

**Fig. 3.**
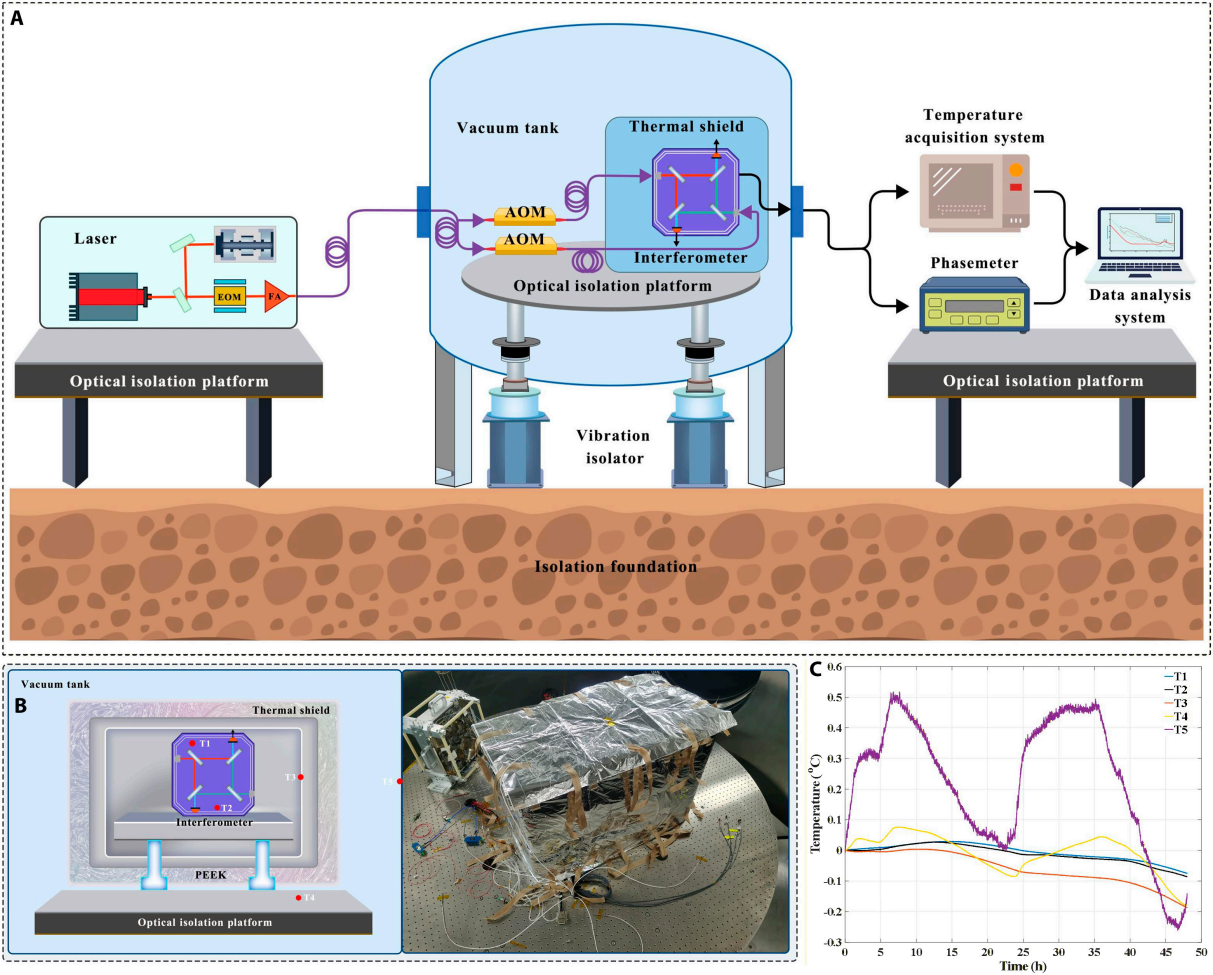
Diagram of the ground-based experimental verification system for the first-generation optical bench of the Taiji program. (A) Overall block diagram of the experimental setup. (B) Schematic and photograph of the thermal insulation cover. T1 to T5 represent temperature sensors located at different positions, including the optical bench (T1: front side, T2: back side), the inner surface of the heat shield (T3), the surface of the optical isolation platform (T4), and the outer surface of the vacuum tank (T5). (C) Temperature fluctuation curve of the experimental system.

The stability of the OB itself is a prerequisite for the attainment of its performance and function. Therefore, in this study, we will initially neglect the other functions and focus solely on testing and evaluating the stability of the OB itself. A ground experimental verification system is designed and built, as shown in Fig. 3. To better evaluate the noise level of the OB under realistic space conditions, it was placed inside a vacuum chamber equipped with an effective vibration isolation system (including an isolation foundation and an isolated optical platform) and a temperature-controlled environment. The chamber was initially evacuated using a mechanical pump. To minimize vibration from the pump affecting the experiment, the pump was shut off once the pressure inside fell below 1 Pa, allowing the chamber to maintain vacuum passively. Moreover, a mirror is attached to the base plate of the OB at the end of the TM interferometer and serves as a replacement for the TM. The difference between the TM interferometer and the reference interferometer in a single OB is used to evaluate the performance of the interferometer system.

As shown in Fig. 3A, a stable laser beam (SLS-1064-300-1000) enters the vacuum chamber via a fiber optic flange and is subsequently split into 2 parts by a fiber optic beam splitter (BS). These 2 beams undergo frequency shifting through an AOM (acousto-optic modulator), resulting in 2 beams with a specific frequency difference (1.6 MHz); this is called the heterodyne frequency. These beams are then connected to 2 fiber collimators located on the OB of the interferometer. After interferometric signals are formed, they are converted into analog voltage signals by a quadrant photodetector positioned on the back side of the OB. These signals are transmitted outside the vacuum chamber through radio frequency cables and flanges and are fed into a phasemeter for phase calculation [33].

The main noise source of the interferometer at low frequencies, especially below 10 mHz, is the thermal drift noise. The experimental system operates at room temperature. To further enhance the thermal stability, a thermal insulation cover is placed on the optical isolation platform within the vacuum tank, as depicted in Fig. 3B. This cover consists of a shell lined with an aluminum plate, an aluminum platform for housing the interferometer, and a thermal insulation layer enclosing the outer shell. The thermal conductivities of the aluminum plate and the invar optical platform are 237 W/(m·K) and 13.7 W/(m·K), respectively. Employing a polyetheretherketone (PEEK) gasket with a thermal conductivity ranging from 0.2 to 0.3 W/(m·K) between the optical platform and the thermal insulation cover effectively mitigates the impact of the external temperature fluctuations on thermal conduction. The insulation layer serves to block the external radiation and maintains the temperature stability within the thermal insulation cover.

To verify the suppression effect of the thermal shield, multiple temperature sensors are affixed to various locations, as indicated by the red dots in Fig. 3B. PT100 platinum resistance sensors are used, and they are directly affixed to the surface of the object being measured using adhesive tape. The acquisition system (Vtest-1101x) has a temperature measurement resolution of 0.001 °C, an accuracy within ±0.010 °C, and a sampling frequency that can reach 0.2 Hz. The temperature fluctuation curve of the experimental system after the vacuum tank has been stabilized for 1 d following vacuum extraction is shown in Fig. 3C. The outer surface of the vacuum tank clearly experiences a daily cycle of temperature fluctuations, which consequently impacts the temperature environment of the OB surface. The temperature drift gradually decreases from the outside of the vacuum tank to the inside and ultimately to the inside of the thermal shield. This trend highlights the effectiveness of the thermal shield in mitigating the temperature drift.

### Noise analysis

**Fig. 4.**
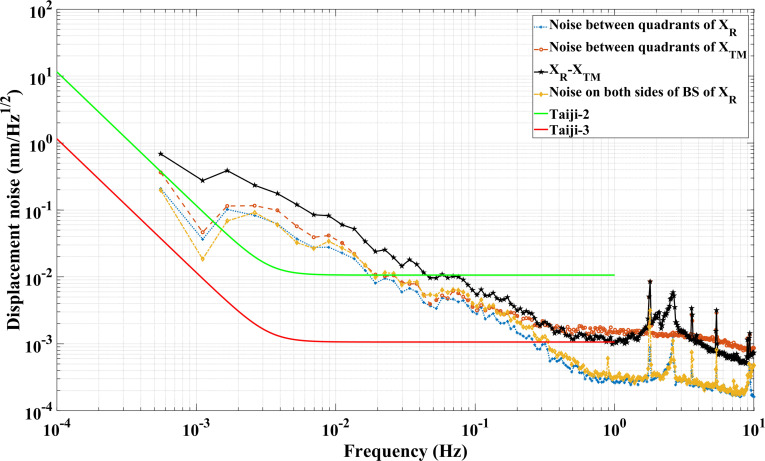
Preliminary test results of the interferometer system under vacuum. X_R_ and X_TM_ represent the reference interferometer and the TM interferometer, respectively.

After the environment stabilized, experimental data were collected and analyzed. A typical experimental result is shown in Fig. 4. The noise between the quadrants reflects the level of the beam pointing jitter and can also be considered the optimal noise level for measurements when the jitter is disregarded. The noise on both sides of the BS is also derived from any quadrant of the QPDs located at those ends. Therefore, it can be analogously compared to the noise between the quadrants, with the addition of noise introduced by the different detectors. Under conditions where the detector noise is minimal, the noise between the quadrants is equivalent to the noise on both sides of the BS. The noise among the different interferometers, which integrate all sources of noise, reflects the overall noise level of the interferometer system and serves as the ultimate indicator for noise evaluation in this study.

Figure 4 shows that the interquadrant noise of the reference interferometer maintains a noise level of 0.3 pm/Hz^1/2^ at 1 Hz and exhibits a 1/f trend at lower frequencies, reaching 0.1 nm/Hz^1/2^ at 1 mHz. The noise on both sides of the BS has the same magnitude and trend, indicating that the noise between the QPDs can be neglected. The difference between the reference interferometer and the TM interferometer is limited to the signal-to-noise ratio of the TM interferometer at 1 Hz, with a noise level of 1 pm/Hz^1/2^. At low frequencies, the sensitivity curve can reach 6 pm/Hz^1/2^ near 0.1 Hz, 70 pm/Hz^1/2^ at 0.01 Hz, and 300 pm/Hz^1/2^ at 1 mHz and exhibits 1/f behavior. However, the overall noise is still large and needs further optimization to meet the requirements of Taiji-2 and Taiji-3. A detailed noise discussion and suppression are analyzed below.

#### Laser power fluctuation

The impact of laser power fluctuations is shown by 2 main aspects [34]: (a) Low-frequency power perturbations (below 1 Hz) cause fluctuations in the radiation pressure on the surface of the free-floating TM, introducing additional force perturbation noise. The stability requirement for optical power in this frequency band is 10^−4^ /Hz^1/2^. (b) High-frequency power perturbations (in the MHz range) are shown as intensity noise coupling with the heterodyne signal, causing additional phase perturbations near the heterodyne frequency and mixing with the measured signal to produce optical path noise. The stability requirement for the optical power in the MHz frequency band is 10^−8^/Hz^1/2^.

**Fig. 5.**
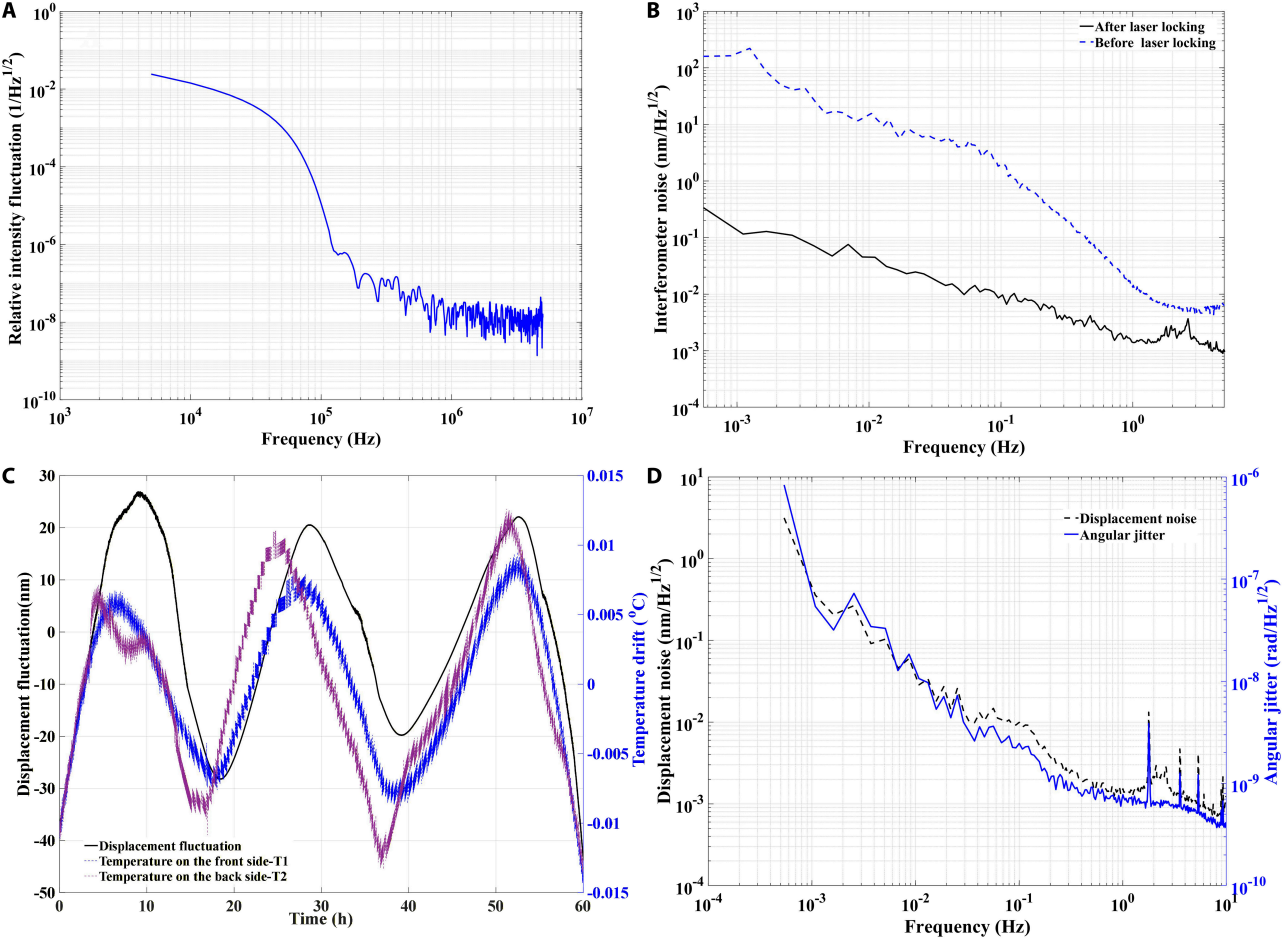
Relevant results of various noise analyses for the optical bench. (A) Spectrum of the relative intensity fluctuation in the MHz frequency range. (B) Interferometer noise performance of the ultrastable laser before and after frequency locking. (C) Temperature drift and optical path fluctuation time domain curves over the 60-h testing period. (D) Frequency spectrum curves of the angular jitter and interferometer noise curves.

For the interferometer system used in this study, the free-floating TM is replaced by a reflecting mirror attached to the breadboard so that the laser radiation pressure noise acting on the TM can be neglected. Therefore, the phase disturbance caused by the intensity noise mainly arises from the second aspect mentioned above. The experimentally measured RIN (relative intensity noise) curve of the laser in the MHz band is shown in Fig. 5A.

From Fig. 5A, the intensity stability of the laser is approximately 3 × 10^−8^/Hz^1/2^ in the frequency band of MHz. From previous research, the RIN noise is calculated by the following equation [34]:

$$\varphi_{1f-\mathrm{RIN}}=\frac{P_{m}+P_{r}}{\sqrt{2\eta_{\mathrm{het}}P_{m}P_{r}}}\tilde{r}\left(1f_{\mathrm{het}}\right)$$ (1)

where *P_m_* and *P_r_* represent the intensities of the measurement beam and the reference beam, respectively; $\eta_{\mathrm{het}}$ represents the heterodyne efficiency; and $\tilde{r}\left(1f_{\mathrm{het}}\right)$ represents the relative intensity fluctuation of the laser beam at the first heterodyne frequency. From this equation, RIN is related to the intensity ratio and the relative intensity fluctuation of 2 interfering beams. In this study, the intensity ratio is controlled within 1:3. Therefore, the RIN is less than 10^−6^ rad/Hz^1/2^, which can be ignored in this study.

#### Laser frequency jitter

The laser frequency noise *δl* is related to the frequency stability of the laser beam and the length difference of the interferometer arms, which can be written as [35,36]

$$\delta l=\frac{\delta f}{f_{0}}\Delta L$$ (2)

where *δf* and *f*_0_ represent the laser frequency jitter and the laser frequency, respectively, and Δ*L* represents the arm length difference of the interferometer. In this work, the TM is replaced by a reflector attached to the OB, significantly increasing the unequal arm length of the TM interferometer. The reference interferometer arm length difference is 13 mm, and the TM interferometer arm length difference is 378.67 mm. Therefore, the difference in arm length between the reference and TM interferometers is 365.67 mm. The frequency stability of the ultrastable laser after locking is approximately 550 Hz/3 h, corresponding to a frequency noise of 0.7 pm/3 h. The laser frequency stability requirement can meet the application measurement requirements. To verify the analysis, we measured the interferometer noise performance of the ultrastable laser before and after frequency locking, as shown in Fig. 5B.

Figure 5B shows a considerable improvement in noise performance before and after laser frequency locking. Through the PDH (Pound–Drever–Hall) cavity locking technique, the laser frequency noise can be effectively suppressed, especially below 0.1 Hz. However, after the frequency noise is suppressed, the curve does not reach a very low level. Evidently, the laser frequency noise is not the main source at this point, and other noises, such as thermal drift noise, angular jitter noise, electronic noise (photodetector, phasemeter), and vibration noise, become dominant.

#### Thermal drift

Although a thermal shield is present outside the interferometer and the thermal shield is protected by a vacuum tank, the temperature drift noise is still the main factor limiting the measurement sensitivity of the interferometer at low frequencies. While laser interferometers with an equal-arm design (e.g., LISA Pathfinder) effectively suppress common-mode noise, the OB in this study has an unequal arm length of 365.67 mm due to the mirror substitution for the TM, thus further amplifying thermal drift noise. Two main methods are used to reduce thermal drift noise: stabilizing the temperature environment and post-data processing. The former method mainly improves the thermal stability of the environment where the OB is located through further temperature control; however, this method is costly and not the focus of this study. The latter method involves analyzing the relationship between the temperature and displacement drift to obtain the temperature coupling coefficient and then eliminating the thermal drift noise through post-data processing. When the measurement environment cannot meet the requirements, post-data processing becomes an effective means. Therefore, in this study, the DCP (disturbance measurement–correlation analysis–post data suppression) method is adopted to improve the measurement sensitivity of the interferometer [37,38].

The displacement error Δ*s* caused by the temperature fluctuations in the beam of a laser propagating through optical materials can be expressed by the following equation [39]:

$$\frac{d\Delta s}{\mathrm{dT}}=\frac{L}{\sqrt{1-\frac{1}{2n^{2}}}}\cdot \left(\alpha \cdot n+\frac{\mathrm{dn}}{\mathrm{dT}}\cdot \left(1-\frac{n}{2n^{3}-n}\right)\right)$$ (3)

where *T* represents the temperature, *n* represents the refractive index, *L* represents the physical length of the beam propagation, *α* represents the CTE (coefficient of the thermal expansion), and *dn*/*dT* represents the coefficient of the refractive index temperature. The displacement noise caused by temperature is related to the physical length, refractive index, CTE, and coefficient of the refractive index temperature, and the last 3 parameters also vary with temperature.

First, we estimate the magnitude of this value based on a simple optical lens. In this study, the optical lens is constructed from Corning 7980, which has a refractive index of approximately 1.45, a temperature refractive index coefficient of approximately 9.6 × 10^−6^/K, and a CTE of 0.52 × 10^−6^/K from 5 to 35 °C. When a laser beam incident at a 45° angle passes through a lens with a thickness of 10 mm, the coupling coefficient between the optical path noise and temperature noise is approximately 8.4 × 10^−8^ m/K [37]. In the Taiji program, the OB of the interferometer is composed of multiple functional lenses and a Zerodur baseplate (the CTE is less than 10^−7^/K), causing difficulty to accurately calculate this value. In this study, the refractive index, CTE, and temperature-dependent refractive index coefficient can be considered constants within a small temperature range. Therefore, the DCP method can be used to decrease the thermal drift noise.

The temperature drift and optical path fluctuation time domain curves over the 60-h testing period are shown in Fig. 5C. To better illustrate the fluctuation patterns of these physical quantities, the data are processed using detrended fluctuation analysis with first-order trend removal.

Over a longer period, the temperature exhibits notable periodic changes, indicating a diurnal cycle with daily variations. These results indicate that the temperature disturbance arising from the vacuum thermal shield is influenced by the external environmental thermal disturbances, leading to identical fluctuations in the optical path curve. These phenomena show a strong correlation between the temperature drift and optical path fluctuations at low frequencies.

**Fig. 6.**
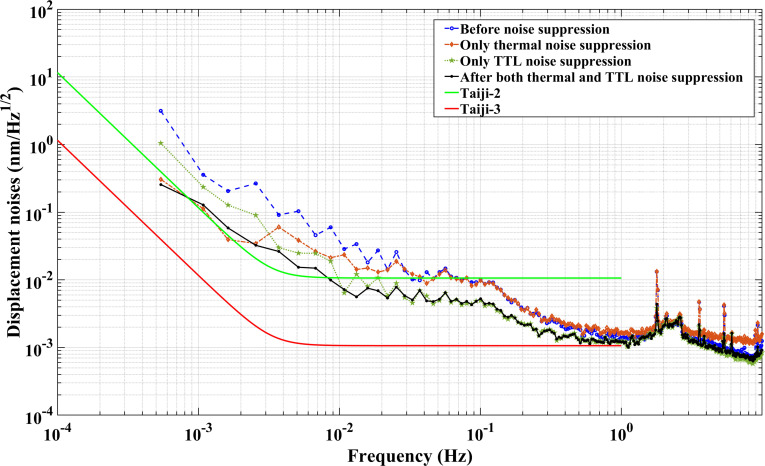
Typical noise suppression effect after the thermal drift noise and TTL noise suppression.

As mentioned earlier, when the ambient temperature fluctuations cannot be effectively suppressed further, the DCP method is employed to further mitigate the thermal drift noise. First, using temperature sensor T1 as a reference, we perform curve fitting on the temperature *T* and the displacement fluctuation *y* via the least squares method. Given that parameters such as the temperature coefficient of the refractive index vary with temperature, we adopt a quadratic fit to account for the coupling between the temperature and displacement. Second, by incorporating the original temperature data into the fitted curve, we can derive the optical path noise *y*(*T*) resulting from thermal drift. Finally, by subtracting the thermal drift noise-induced optical path noise *y*(*T*) from the original optical path data *y*, we obtain the noise performance of the interferometer system *y*-*y*(*T*) after the thermal drift noise is suppressed. Figure 6 (dark red dash-dot curve) shows a typical sensitivity curve after the thermal drift is suppressed.

Figure 6 shows that the sensitivity curve is effectively improved by suppressing the thermal drift noise in the frequency band below 0.01 Hz. Prior to and following the suppression, the noise curve at 1 Hz exhibits no notable change, remaining below 2 pm/Hz^1/2^. The primary limiting factor is the electronic noise, which can be reduced by the further enhancement of the interferometer’s intensity. Similarly, the noise curve at 0.1 Hz remains unchanged and is approximately 10 pm/Hz^1/2^. At 10 mHz, the noise level is reduced approximately 2-fold and is 20 pm/Hz^1/2^. Notably, the noise suppression effect at 1 mHz is remarkable, improving by approximately a factor of 5 with respect to that at 100 pm/Hz^1/2^. However, as shown in Fig. 6, the noise curve after thermal noise reduction still fails to meet the requirements of Taiji series missions. This finding indicates that thermal drift noise is not the dominant factor at this point, and further analysis and noise suppression are needed, particularly in the frequency band below 0.1 Hz.

#### Beam pointing jitter

In the interferometer system, the laser beam is introduced to the OB through optical fibers and converted into spatial light by fiber collimators, which then form various functional spatial light paths. Due to environmental disturbances such as vibration and temperature, the pointing angle of the collimated beam changes. Owing to technological limitations, the fiber collimators used in this study are not self-developed all-glass structures connected to the optical platform using HCB (hydrogen–oxygen catalytic bonding) technology. Instead, traditional commercial collimators are adopted, which are bonded to the base plate using epoxy resin with an Invar-structured clamp (FIOS). This structure intensifies the changes in the beam pointing angle caused by the environmental disturbances. Moreover, in addition to the disturbances caused by the collimator, environmental perturbations to the optical mirrors, such as the reflectors and beamsplitters, can also lead to changes in the pointing angle of the beam. This change in the pointing angle of the beam generates the so-called TTL (tilt-to-length) noise [40,41], which is currently a popular topic in interferometer noise research.

The causes of the TTL noise can be divided into geometric effects and nongeometric effects. Geometric effects describe the additional displacement caused by changes in the actual optical path when the beam angle changes. Geometrically, this effect can be further divided into the lever effect and the piston effect. The former describes the increase in the actual optical path caused by changes in the beam angle, whereas the latter describes the additional optical path generated when the rotation center of the optical element is not in the ideal position. Nongeometric effects describe the mismatch of the wavefronts between the 2 interfering beams, resulting in additional wavefront errors due to angular changes. In practice, the lever effect can be eliminated by the lens group (primarily functioning on the back of the OB); however, the piston effect can currently be suppressed only by the precise adjustment of the rotation center to its ideal position. Nongeometric effects are generally nonlinear high-order quantities that can be disregarded relative to the geometric effects. Therefore, in this work, the TTL noise is generated mainly by the piston effect. If the angular fluctuation of the photodetector is neglected, the TTL noise Δ*s* can be simply represented by the following equation [42]:

$$\Delta s=-2d_{\mathrm{lat}}\cdot \text{cos}\left(\beta \right)\cdot \varphi +\left(2d_{\mathrm{lat}}\cdot \text{sin}\left(\beta \right)+d_{\text{long}}\cdot \text{cos}\left(\beta \right)\right)\cdot \varphi^{2}$$ (4)

where β and φ represent the static incident angle of the beam and its pointing jitter, respectively, and *d*_lat_ and *d*_long_ refer to the horizontal and vertical components of the rotation of the optical component around the rotation center, respectively.

The above equation shows that the coupling coefficient varies nonlinearly for different static angles β. For small angular jitter (below μrad) around a fixed static angle deviation, the coupling coefficient varies linearly, as the quadratic term in its expansion is negligible. The influence of this quadratic term, however, becomes ‌non-negligible‌ only when the angular jitter is large. In previous fittings of the temperature coefficient, we employed a time-domain fitting approach. Conversely, if the coupling coefficient proves difficult to extract accurately in the time domain and the physical quantity is frequency-independent, one can perform a direct fit within a correlated frequency band instead. Therefore, we adopt a frequency-domain fitting method here [37]. The spectra of the angular jitter and interferometer noise curves are shown in Fig. 5D.

Figure 5D shows good consistency between the angle jitter and displacement noise between 0.1 and 0.005 Hz; thus, the noise in this frequency band is caused mainly by the TTL noise. We can obtain the coupling coefficient by using the value at a frequency of 0.1 Hz in the following equation:

k=Δsφ≈2.69pm/nrad (5)

After the coupling coefficient is obtained, the influence of the angle jitter coupling noise, *k*·*φ*, is subtracted from the initial reference–TM displacement noise *y*, and the noise suppression effect is observed. The typical result after the TTL noise is suppressed is shown by the dark green dotted curve in Fig. 6.

Within the frequency range of 3 mHz to 0.4 Hz, the noise curve can be significantly enhanced by suppressing the TTL noise. Prior to and following the suppression, the noise curve at 1 Hz remains largely unchanged and is superior to that at 2 pm/Hz^1/2^. At 0.1 Hz, the noise level is reduced by approximately 2 times and outperforms that at 5 pm/Hz^1/2^. The noise suppression effect is particularly pronounced at 10 mHz, and the noise level decreases by approximately 3 times, surpassing 10 pm/Hz^1/2^. However, the noise suppression effect at 1 mHz is less evident. Consequently, the suppression of the TTL noise is more notable in the frequency band of 3 mHz to 0.1 Hz; however, the noise suppression in the lower mHz frequency range is not satisfactory.

The above analysis reveals that the thermal drift noise and TTL noise affect different frequency bands. Therefore, to obtain better results, we attempt to jointly eliminate both noise types. The crucial point in simultaneously eliminating both noises is their decoupling because thermal drift can also cause angular jitter. When the thermal drift noise is eliminated, the temperature-induced component of angular jitter is also eliminated. Therefore, when the angular jitter is eliminated, the original angular fluctuation information cannot be used. Consequently, the following approach is adopted to eliminate both types of noise. First, the thermal drift noise is removed from the original data, as described previously. Second, the temperature-induced component of angular jitter is reduced, leaving only vibration-induced angular jitter. This process decouples the thermal drift and angular jitter. Finally, the angular coupling coefficient is obtained through the corrected angular information to eliminate the vibration-induced TTL noise. A typical result is shown by the black solid curve in Fig. 6.

As shown in the figure, after both the thermal drift noise and TTL noise are suppressed, the noise curve exhibits a significant suppression effect across the various frequency bands, and a maximum reduction of one order of magnitude with respect to the initial optical path noise is attained. At 1 Hz, the primary limiting factor is electronic noise, which is reduced from 1.5 pm/Hz^1/2^ to 1.1 pm/Hz^1/2^ following mitigation. The main approach to further reduce this noise is to increase the laser intensity of the interferometer. At 0.1 Hz, the primary limiting factor is the TTL noise, which is reduced from 10 pm/Hz^1/2^ to 5 pm/Hz^1/2^ after mitigation. At 0.01 Hz, the main limiting factors are the TTL noise and thermal drift noise; these noise types are reduced from 40 pm/Hz^1/2^ to 8 pm/Hz^1/2^ following mitigation. At 1 mHz, the main limiting factor is the thermal drift noise, which is reduced from 500 pm/Hz^1/2^ to 150 pm/Hz^1/2^ after mitigation.

#### Other noises

Based on the discussion and suppression of the abovementioned noise, particularly the inhibition of thermal drift noise and angular jitter noise, the current noise level of the interferometer system can be suppressed by approximately one order of magnitude in the frequency range of 0.01 to 1 Hz, which meets the requirements of the Taiji-2 mission. However, the noise level has not yet reached the final requirements of the Taiji program. As summarized in Table 3, we present the results before and after noise suppression in this paper, along with an analysis of the dominant noise sources identified in the expected results and the corresponding suppression methods employed.

**Table 3.** Summary of noise characterization and mitigation for the first-generation optical bench of the Taiji program

Considered cases	1 mHz	0.01 Hz	0.1 Hz	1 Hz
Preliminary results (limited noises)	500 pm/Hz^1/2^ (thermal drift)	40 pm/Hz^1/2^ (TTL, thermal drift)	10 pm/Hz^1/2^ (TTL)	1.5 pm/Hz^1/2^ (electronic noise)
After mitigation (limited noises)	150 pm/Hz^1/2^ (thermal drift, stray light)	8 pm/Hz^1/2^ (OPD, stray light, TTL)	5 pm/Hz^1/2^ (OPD, TTL)	1.1 pm/Hz^1/2^ (electronic noise)
Expected results (suppression scheme)	10 pm/Hz^1/2^ (more stable temperature control, balanced detection)	1 pm/Hz^1/2^ (front-end optical path locking, balanced detection)	1 pm/Hz^1/2^ (front-end optical path locking, all-glass FIOS utilizing HCB)	1 pm/Hz^1/2^ (increase the optical power or interference efficiency)

From Table 3, to further reduce the noise level, research will be carried out in the following areas: (a) optical path-length difference (OPD) noise [43,44]. The final noise curve in Fig. 6 shows the presence of a “shoulder” shape within the frequency range of 0.01 to 0.1 Hz. This shape may be due to the OPD noise generated by the AOM sideband. To address this, an active control method of front-end optical path locking using piezoelectric transducer (PZT) will be adopted to reduce the impact of OPD noise. (b) Stray light [45]. Stray light, referring to parasitic interference caused by nontarget light, can be suppressed either by physically blocking it from the detector path or through balanced detection. The latter uses 2 detectors on either side of the beamsplitter; since the stray light noise is identical in both detectors but the main signal is opposite in sign, subtracting the 2 signals cancels the noise. (c) Thermal drift noise. The current suppression of thermal drift noise is relatively coarse due to hardware limitations, primarily reflected in the uneven temperature distributions at different locations and the limited precision of the temperature sensors. However, considering both the low CTE of the optical platform (10^−7^/K) and the sufficiently small temperature gradient (the difference between T1 and T2 in Fig. 3 is far less than 0.1 K), this study adopts the single-position temperature fitting approach. To further reduce temperature drift noise, higher-precision, multipoint temperature sensing and active temperature control will be essential. (d) TTL noise. Currently, the reduction in TTL noise is accomplished by one-direction suppression, whereas vibrations may be multidirectional. Eliminating TTL noise caused by multidimensional vibration is one of the tasks for the next stage of research. While TTL noise is projected to be a major source of optical path noise in future Taiji interferometers, primarily due to angular jitter of the TM or intersatellite laser beams, the dominant source identified in this work originates from the structural instability of the FIOS. This instability causes jitter in the outgoing beam. The primary strategy for resolving this issue is to advance the development of an all-glass FIOS utilizing HCB to enhance the pointing stability of the output beam in the future.

## Conclusion

In this study, we have designed a full-function interferometer OB for the Taiji program and, for the first time, provided detailed parameter specifications for its interferometer system. Moreover, an experimental system is established with the aim to conduct noise testing and calibration on the first-generation OB of the Taiji program. To reduce the measurement noise of the interferometers, the laser intensity noise, laser frequency noise, thermal drift noise, and angular coupling noise of the system were thoroughly analyzed, leading to the establishment of detailed noise models. The analysis revealed that the first 2 noise sources did not substantially affect the system, whereas thermal drift noise and TTL noise were identified as the main factors influencing the optical path noise. By employing the DCP method, 2 types of optical path noise were specifically processed and suppressed within the designated frequency bands. As a result, the interferometric measurement accuracy improved by approximately one order of magnitude compared with that before noise suppression. Notably, the sensitivity curve achieved 8 pm/Hz^1/2^ at 0.01 Hz, 5 pm/Hz^1/2^ at 0.1 Hz, and 1.1 pm/Hz^1/2^ at 1 Hz; thus, these results met the requirements of the Taiji-2 mission.

This work marks the first step in transitioning the Taiji program’s interferometer system from a principle prototype to an engineering prototype, playing a critical role in its future development. To further meet the requirements of the Taiji program across the entire frequency range, the interferometer system will be further optimized by addressing noise reduction issues such as the OPD noise, stray light, more precise suppression of thermal drift noise, and elimination of angular coupling noise. In parallel, we will conduct a series of environmental tests on the optical bonding technology and the OB to verify their engineering reliability. These tests will include thermal cycling, thermal vacuum, and vibration testing, ensuring that the OB meets the stringent requirements for future space applications within the Taiji program.

## Materials and Methods

Unlike the LISA Pathfinder, the OB of the Taiji mission features a 3-dimensional structural design. Compared with the planar structure, the 3-dimensional structure involves the transformation of the reference coordinate system, which makes the transmission of angular errors more difficult. The front side is mainly composed of nonheating optical components, such as fiber collimators, beamsplitters, mirrors, and wave plates. The back side is mainly composed of the detection devices, such as CCDs and photodetectors. This design can effectively reduce the impact of heat on the optical lenses on the front side.

**Fig. 7.**
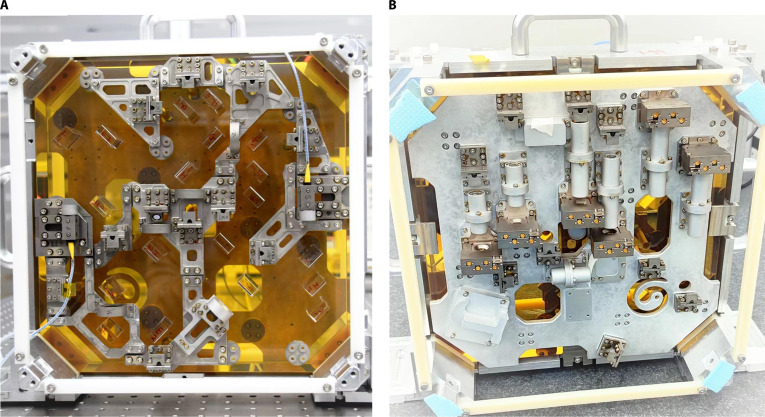
Photographs of the first-generation optical bench of the Taiji program: (A) front side and (B) back side.

To construct a high-strength, low-stress OB, a Zerodur (Schott) with a length of 320 mm, a width of 320 mm, and a thickness of 70 mm was used as the baseplate. Dozens of fused silica (Corning 7980) glass blocks, which are 20 mm long, 10 mm wide, and 35 mm high, are used as optical components and are coated with various optical properties of dielectric films. The Zerodur baseplate and the fused silica glass blocks are bonded together by using HCB technology [46–48], and the bonding surfaces for HCB are polished to a root mean square (RMS) value of less than λ/10 (λ = 632.8 nm). Due to the immaturity of relevant technologies, the OB of Taiji’s first generation still adopts commercial-grade fiber collimators (OZ Optics, LPC-04-1060-6/125-P) on the front side. The optical substrate is bonded to an invar bracket, and the PAAM is also bonded to the optical substrate using an invar bracket. The remaining optical lenses are connected using HCB optical bonding technology. After interference on the front side, the laser beam is reflected to the back side through a right-angle mirror for relevant detection. The back side mainly includes detectors and a BSMG (beam shrinking mirror group), which are bonded to the substrate using invar devices. A photograph of the first-generation OB of the Taiji program is shown in Fig. 7.

## Data Availability

The data that support the findings of this study are available from the corresponding author upon reasonable request.
